# A Novel Three-Filament Model of Force Generation in Eccentric Contraction of Skeletal Muscles

**DOI:** 10.1371/journal.pone.0117634

**Published:** 2015-03-27

**Authors:** Gudrun Schappacher-Tilp, Timothy Leonard, Gertrud Desch, Walter Herzog

**Affiliations:** 1 Department of Mathematics and Scientific Computing, University of Graz, 8010 Graz, Austria; 2 Human Performance Laboratory, University of Calgary, Calgary, Alberta, Canada; Semmelweis University, HUNGARY

## Abstract

We propose and examine a three filament model of skeletal muscle force generation, thereby extending classical cross-bridge models by involving titin-actin interaction upon active force production. In regions with optimal actin-myosin overlap, the model does not alter energy and force predictions of cross-bridge models for isometric contractions. However, in contrast to cross-bridge models, the three filament model accurately predicts history-dependent force generation in half sarcomeres for eccentric and concentric contractions, and predicts the activation-dependent forces for stretches beyond actin-myosin filament overlap.

## Introduction

The generally accepted mechanism of active force production in a sarcomere is based on a pioneering model by [1], the so-called cross-bridge theory. Briefly, myosin heads attach to the actin filament and pull the actin filament towards the M-line in the centre of the sarcomere. Thereby the sarcomere shortens and produces active force. Main determinants of active force productions are the contraction velocity as well as the overlap of actin and myosin filaments which is a function of sarcomere length.

The great success of the cross-bridge model is based on nearly flawless predictions of contractions at constant sarcomere length (isometric contractions) and contractions where the sarcomere is allowed to shorten (concentric contractions). However, it cannot account for some prominent observations when an activated sarcomere is stretched (eccentric contractions). One such observation is the so-called residual force enhancement. After an active stretch of a muscle unit steady-state forces are increased compared to the corresponding isometric forces thereby contradicting the force-length relationship predicted by the cross-bridge model.

In addition, recent experiments on single myofibrils prepared from rabbit psoas [2] revealed another powerful mechanism of active force production. Myofibrils were activated near optimal myosin-actin overlap (∼2.4 μm/sarcomere) and stretched along the descending limb of the force-sarcomere length relationship to final lengths of up to 6 μm/sarcomere. At lengths of around 4 μm/sarcomere, the overlap between thin and thick filaments is lost [3]. Therefore, the cross-bridge model predicts zero active forces for sarcomere lengths beyond 4 μm. Thus, when stretched beyond actin-myosin overlap, forces are thought to be caused by passive structural elements only and merely a function of muscle length [4]. However, experiments revealed a different behaviour. Forces, corrected by subtracting passive forces, continue to rise when sarcomeres are stretched beyond myofilament overlap and exceed the maximal active forces at optimal myofilament overlap by more than 200%. Therefore, there has to be a powerful force generating mechanism at long sarcomere lengths that does not rely on actin-myosin based cross-bridge interactions.

In myofibrils passive force production is governed virtually exclusively by the behaviour of the giant protein titin [5,6] which spans the half sarcomere from Z disk to M band. A part of titin’s region located in the I-band segment of sarcomeres functions as a molecular spring that elongates when sarcomeres are stretched, thereby developing passive forces. It is well accepted that titin alters its properties in the presence of calcium, by binding calcium in the so-called PEVK areas and some immunoglobulin (IG) domains (e.g. [7–10]). However, these alterations are insufficient to explain the massive gain of force at long sarcomere lengths [11].

**Fig 1:**
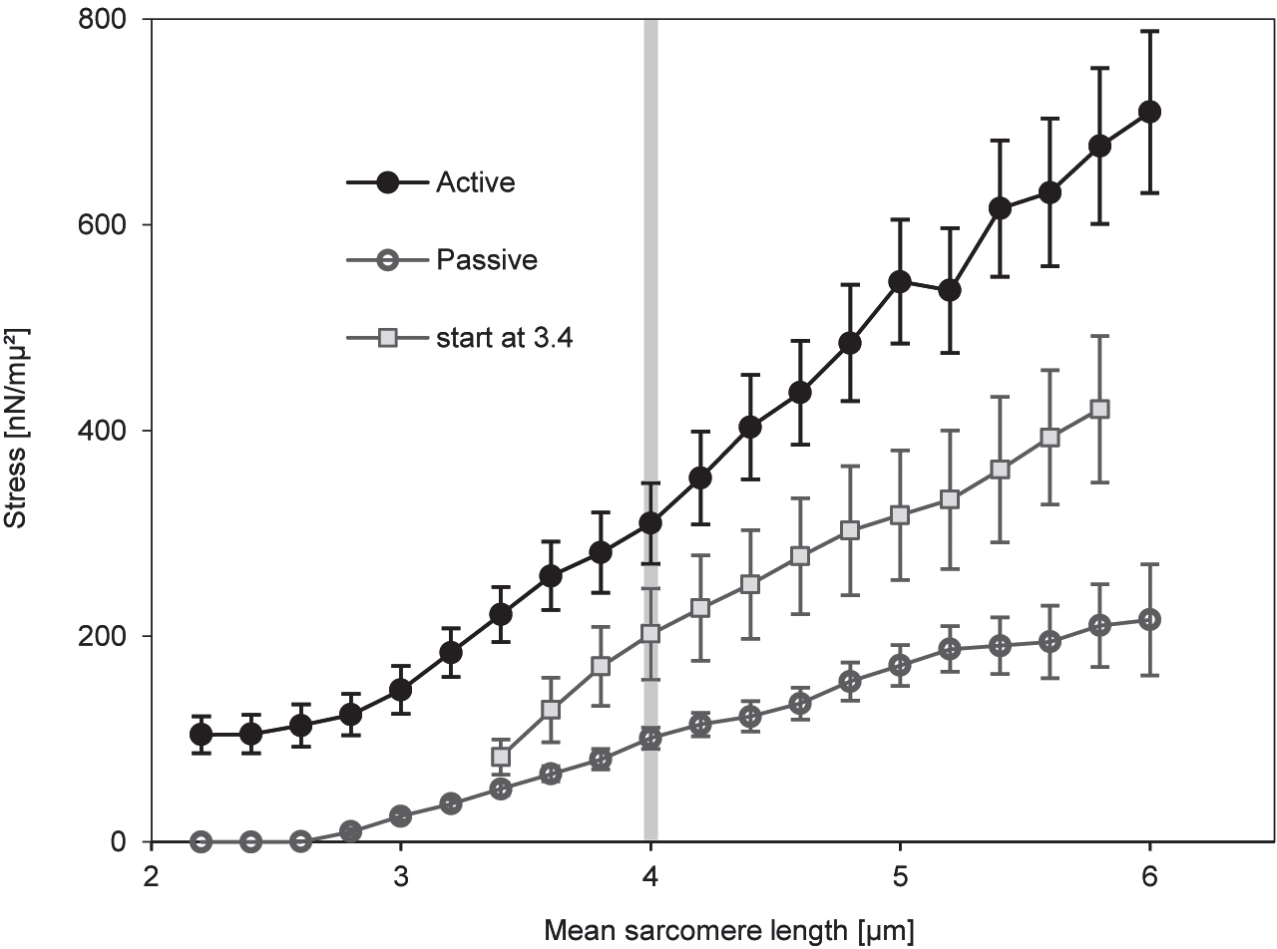
Mean ± standard deviation of stress versus average sarcomere length of myofibrils. Myofibrils were stretched in a low-calcium solution (passive stretch, open circles) and high-calcium solution from an initial sarcomere length of 2.4μm (active stretch, black circles) and 3.4μm (active stretch from 3.4μm/sarcomere, grey squares). Based on the cross-bridge model, forces beyond actin-myosin filament overlap (sarcomere length > 4μm; grey vertical bar) in actively stretched myofibrils are predicted to coincide with forces in passively stretched myofibrils. However, forces in actively compared to passively stretched myofibrils are three to four times higher when the stretch starts at an initial sarcomere length of 2.4μm, and around twice as high when the stretch starts at an initial sarcomere length of 3.4μm. Careful experimental and theoretical testing and analysis of all individual sarcomere lengths revealed that these results cannot be explained by the development sarcomere length non-uniformities. A detailed description of the experiments is given elsewhere [2].

Moreover, the force generating mechanism beyond actin-myosin overlap is history dependent [11]. Myofibrils stretched passively to 3.4 μm/sarcomere and then activated and stretched to 6 μm/sarcomere develop more force than could be explained by the cross-bridge theory and contributions of passive elements. However, forces for these myofibrils stayed well below the forces obtained in myofibrils stretched from optimal filament overlap of 2.4 μm/sarcomere (Fig. 1).

There are several theories that might explain the much greater forces at sarcomere length beyond myofilament overlap for myofibrils stretched actively (high calcium concentration) compared to those stretched passively (low calcium concentration). For example, the winding-filament theory that postulates a winding of titin onto the rotating actin filament [12], or the binding of some proximal segment of titin to actin, thereby reducing titin’s free spring length [13,13–18] could explain these experimental observations [11]. Since there is a strong body of evidence that actin-titin interactions are only enabled when myofibrils are activated [11], the history dependence of the force generating mechanism, and the active force observed when sarcomeres are stretched beyond actin-myosin filament overlap, could possibly be explained by activation of force-dependent interactions of titin with actin [19].

### Purpose and outline of this study

We propose a coarse-graining three filament model in which titin binds to the rigid actin filament, thereby shortening its free spring length upon activation, and contributing more force when stretched compared to the passive stretching of sarcomeres.

First, we introduce the mathematical model in the methods section. In the results section we analyse its predictions in detail and show that titin-actin binding not only provides an intriguingly simple explanation for the powerful force generating mechanism in the absence of actin-myosin overlap, but also provides a framework for understanding history dependence of force generation in a single sarcomere, i.e. phenomena like residual force enhancement and force depression [13,20,21].

## Materials and Methods

### Mathematical formulation of the three filament model in half sarcomeres

The proposed model is based on (i) active force production based on cross-bridge interactions and (ii) passive force production based on the elongation of titin. However, the model also allows for actin-titin binding upon activation whenever cross-bridge interaction is not inhibited, thereby altering titin’s spring length and passive force when stretched. The binding between actin and titin does not generate force per se but directly influences active and passive force generation. The total force exerted by myofibrils is then given by the sum of the active (actin-myosin based) force and the passive (variable titin-based) force. We perform all calculations for single half sarcomeres. All parameters are based on the properties of rabbit psoas.

(i) Active force production is modelled by a 3-state cross-bridge model where myosin heads cycle through three states: a detached state, a pre-power stroke, and force generating state [22]. The corresponding system of partial differential equations can be written as

$$\frac{\partial }{\partial t}\vec{p}\left(t,x\right)+v\frac{\partial }{\partial x}\vec{p}\left(t,x\right)=A\left(x,p_{1}\left(t,x\right),p_{2}\left(t,x\right),p_{3}\left(t\right)\right),$$ (1)

where *p*_*i*_(*t*,*x*), *i* = 1,2, is the probability density of a cross-bridge being in the *i*-th state at time *t* with cross-bridge link length *x*. The vector $\vec{p}\left(t,x\right)=\left(p_{1}\left(t,x\right),p_{2}\left(t,x\right)\right)\prime ,$ comprises the probability densities of the two attached states while *p*_3_ corresponds to the detached state.

Active force based on cross-bridge interaction for a given sarcomere length is given by

$$f_{active}(t)=S∙g\left(\text{overlap}\right)∙\int \sum_{i=1}^{2}\chi_{i}(x)p_{i}\left(t,x\right)dx,$$ (2)

where *g* is a geometry function of actin-myosin overlap, *S* is a scaling factor, and χ_i_(*x*) are elastic force functions. The system of coupled partial differential equations (1) with appropriate initial and boundary conditions is solved numerically. A detailed description of the model is given in [22]. This cross-bridge model accounts for force enhancement by active stretching as well as the dynamic response of cross-bridges to an induced stretch [23–25], although residual force enhancement in a single half sarcomere (e.g. [26,27]) cannot be predicted [28].

(ii) The model of passive force production accounts for the complex structure of titin. The main sequence elements of titin’s I-band region are the proximal (near Z-disk) and distal (near M-line) immunoglobulin (IG) segments containing serially linked IG domains, and the PEVK sequence [29]. In intact sarcomeres distal IG domains are thought to aggregate into non-extensible end filaments [30,31]. Therefore, all distal IG domains are modelled as one non-compliant compartment. Throughout the manuscript we assume that force-elongation traces of folded and unfolded proximal IG domains can be modeled by worm-like-chain (WLC) models [32],

$$f_{IG}=\frac{k_{B}T}{pl}\left(\frac{1}{4{\left(1-\frac{x}{cl}\right)}^{2}}-\frac{1}{4}+\frac{x}{cl}\right),$$ (3)

where *f*_*IG*_ is the force needed to stretch one IG domain to an end-to-end length of *x*. The other model parameters are absolute temperature T, the Boltzmann constant *k*_*B*_, the contour length *cl*, which determines the asymptotic behavior of the force-elongation behavior of the IG domains, and the persistence length *pl*, which scales the force.

Force-elongation behavior of the PEVK segment is best modeled using modified WLC models [33,34] which involve a third parameter, the elastic modulus *K*_0_:

$$f_{PEVK}=\frac{k_{B}T}{pl}\left(\frac{1}{4{\left(1-\frac{x}{cl}+\frac{f_{PEVK}}{K_{0}}\right)}^{2}}-\frac{1}{4}+\frac{x}{cl}-\frac{f_{PEVK}}{K_{0}}\right).$$ (4)

Proximal IG domains are thought to unfold subsequently upon high tension [35–37]. Therefore, unfolding has to be taken into account for stretches far beyond the actin-myosin overlap zone, leading to a stochastic model where the number of folded IG domains is a discrete random variable. We assume a two-state unfolding reaction. One IG domain gains 26nm in length upon unraveling. Again, the force–elongation relationship of unfolded IG domains is well reflected by a WLC model (3) where the contour length and the persistence length are adjusted for the unfolded state.

In order to simulate experiments where a myofibril is stretched to a certain length, we formulate the stochastic model for the length of a half sarcomere *HSL*:

$$HSL=N_{u}^{prox}l_{u}^{prox}+{(N^{prox}-N}_{u}^{prox}{)l}_{f}^{prox}+l^{PEVK}+l^{dist}+d,$$ (5)

where $l_{u}^{prox}$ and $l_{f}^{prox}$ are the lengths of an unfolded and folded proximal IG domain, respectively. *N* ^*prox*^ is the total number of proximal IG domains. The discrete random variable $N_{u}^{prox}$ is the number of proximal IG domains representing the unfolding process. Folding and unfolding rates are force dependent [38]; unfolding forces of proximal IG domains show a weak hierarchical structure [39]. Since the myofibril is not allowed to return to a relaxed state, the refolding rate is extremely small and no refolding takes place. The parameter *d* represents half of the length of the A-band region, which is modeled as a very stiff linear spring. Finally, *l* ^*PEVK*^ and *l* ^*dist*^ are the lengths of the PEVK region and the inextensible end filaments, respectively.

We assume that all compartments act in series, implying that forces in all regions are equal. Thus, for a given half sarcomere length *HSL*, we can solve the model (5) by calculating the end-to-end length of each compartment using (3) and (4) whenever the length of titin’s free I-band region is greater than the slack length. The unfolding process is simulated using Monte-Carlo simulations e.g. [38,39]. Our simulations include the hierarchical structure of unfolding forces [39] by adjusting the width of activation barrier in the force depending unfolding transition [38].

The force developed by a single titin molecule upon extension of a half sarcomere was predicted using the 3,400-kD isoform [40], i.e. the number of proximal and distal IG domains and the number of PEVK residues in rabbit psoas are 50, 26, and 800 respectively [41]. The contour length follows from the maximum length of one IG domain (4.5nm); the contour length of the PEVK region follows from the maximum length of one residue (0.36nm) times the number of residues [41].

**Fig 2:**
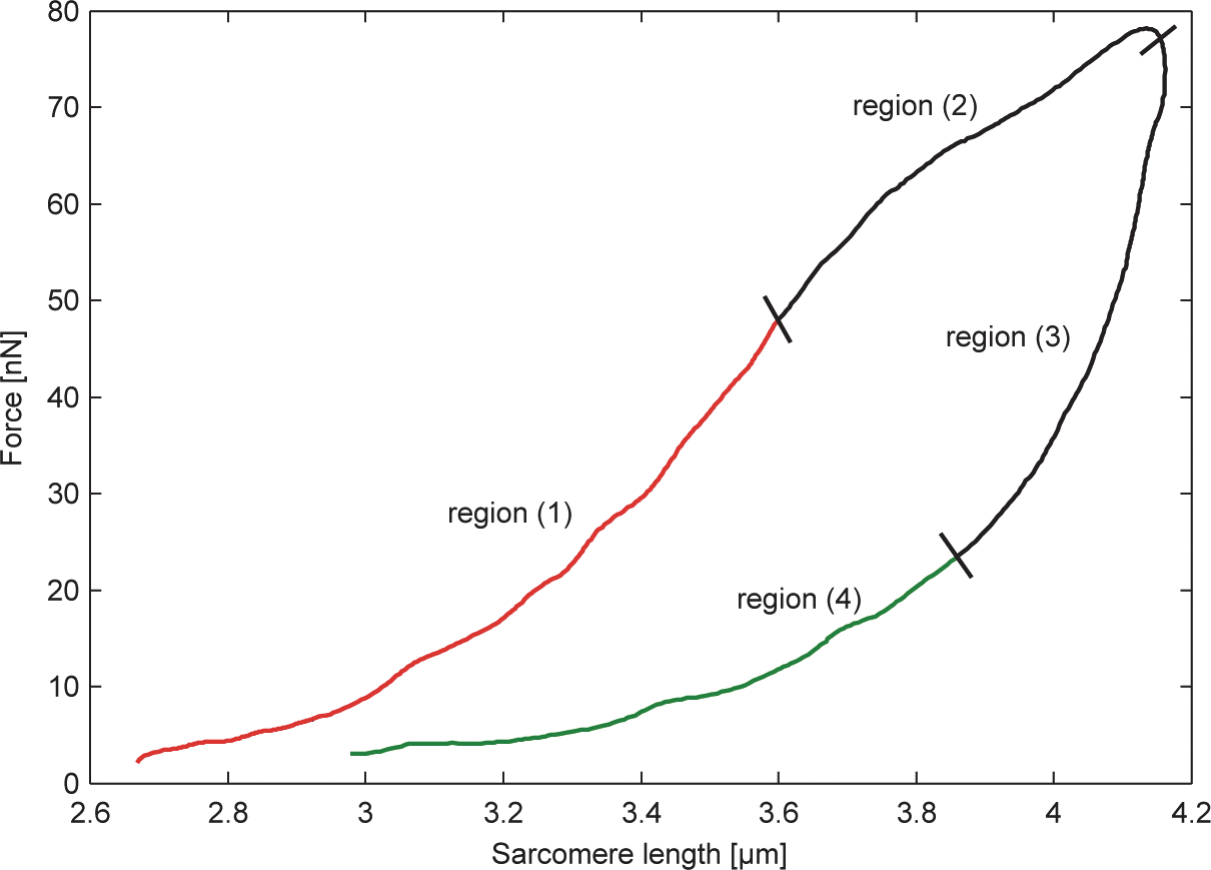
Force versus average sarcomere length of a myofibril first stretched in a low-calcium solution and then shortened again. The red line corresponds to region (1) where no unfolding of IG domains took place; the green line to region (4) where no further folding-unfolding of IG domains took place.

We used independent data based on hysteresis measurements of passively stretched myofibrils [37] for parameter estimation. From the latter study, we could classify hysteresis loops into four parts: (1) from the start of the stretch to an observed inflection point, below which no significant IG domain unfolding processes took place (see also [35]); (2) further stretching led to IG domain unfolding processes; (3) upon shortening of myofibrils, we observed a sharp decrease in force leading to an indefinite state with possible further unfolding of IG domains (despite a decrease in force); and (4) with further shortening of myofibrils, the force-elongation curve followed a WLC model perfectly, suggesting that no further folding-unfolding of IG domains took place in this experimental phase (Fig. 2). We used region (1) to fit the persistence length of folded IG domains, as well as the persistence length and elastic modulus of the PEVK region. After determining these values by a Levenberg-Marquard algorithm, we used region (4) to fit the number as well as the persistence length of unfolded IG domains.

Since it has been shown that titin’s properties are altered upon muscle activation (i.e., for physiologically relevant increases in calcium concentration) resulting in an increase in passive force of about 25% [9], we used a second set of parameters for persistence lengths of IG domains and PEVK region for activated sarcomeres (high calcium concentration).

Furthermore, when the proximal portion of titin binds to the actin filament, its free spring length shortens thereby influencing the stochastic model (5). Although actin is compliant [42] with around 20pm stretch per μm filament length per 1nN/μm^2^ force increase its extension is negligible compared to the extension of titin filaments. We therefore assume that actin is essentially rigid and the actin-titin binding site does not move during half sarcomere stretching.

Finally, we assume that actin-titin interaction is only triggered by an active stretch or shortening introduced by an external force whenever cross-bridge interaction is not inhibited. Thereby, the adjustment of spring stiffness due to the presence of a high calcium concentration is assumed to be instantaneous at the time of activation, while the actin-titin interaction only occurs when the active stretching/shortening occurs. The other extreme scenario reverses this situation by assuming that the actin-titin interaction is independent of an external force and is very fast, thus it occurs first and is only then followed by the calcium binding to titin. Therefore, actin-titin interaction happens instantaneously at the time of activation, while the adjustment of titin’s spring stiffness due to the high calcium concentration is the slower mechanism and occurs only when titin is already bound to actin. The difference between these two scenarios is negligible for sarcomeres activated at a length close to optimal actin-myosin filament overlap. However, it changes isometric forces, if sarcomeres are activated at long length. If not stated otherwise results are based on an instantaneous calcium binding mechanism.

Relative forces are normalized to the corresponding isometric forces. The two scenarios described above describe extremes. In reality, the true mechanism is likely somewhere between these extremes and is based on adjustment rates and attachment rates which need to be quantified in the future.

The location of an actin-titin binding site on the titin filament is in principle a free variable in our model. Since titin has a structure that changes considerably along its length, it is fair to assume that there might only be one distinct site that can bind to the actin filament. There is evidence that titin’s PEVK region binds to the actin filament [16,17,41,43,44]. We chose a conservative approach and assumed that titin binds to actin at the most proximal PEVK residue. Therefore, in the activated state actin-titin binding leaves only the PEVK region as free spring element. Furthermore, we assumed that the actin binding site is variable and that titin binds to the nearest actin binding site, i.e., the length of titin’s compartments is nearly unchanged by the binding process before an active stretch. Therefore, the length of titin‘s proximal portion before the binding determines the force gain due to actin-titin binding during stretch.

All results are based on simulations of a single half sarcomere. Monte Carlo simulations were used for 500 titin strands, and normalized to the passive forces in a single half sarcomere of 1μm^2^ cross section.

## Results

### Force generation in the absence of cross-bridge interaction

**Fig 3:**
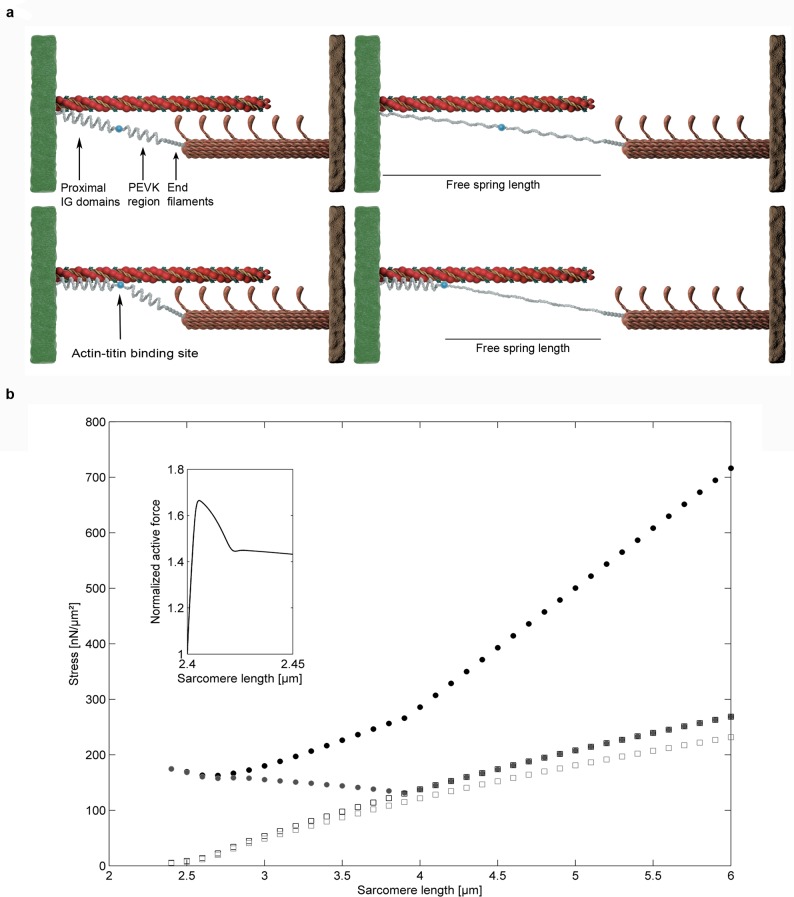
Simulation of stretching a single sarcomere beyond actin-myosin overlap. **a**, Sketch of an active stretch of a sarcomere without actin-titin interaction and with actin-titin interaction. **b**, The sarcomere is activated at a length of 2.4μm and stretched actively until the first protein structures start to break. The solid grey circles show the force-elongation relationship based on active force production by cross-bridges and passive force production based on increased titin stiffness and increased IG domain unfolding forces associated with high calcium concentrations, the open grey squares represent the purely passive forces, while the open black squares represent force production based on increased titin stiffness and increased IG domain unfolding forces but inhibited cross-bridge interaction. Finally, the solid black circles represent an active stretch condition involving actin-titin interactions. The small insert shows normalized active force and thereby the dynamic response of cross-bridges to the induced stretch at higher time resolution.

Due to actin-titin binding at the start of activation, we predict a massive force generation in the absence of cross-bridge interaction. It is worth pointing out that force predictions of half sarcomeres especially at low sarcomere lengths will not scale one-to-one to forces in myofibrils, muscle fibers or whole muscles. Sarcomere non-uniformities as well as intermyofibrillar links change the qualitative behavior of force predictions [45]. In particular the dip of force in the region of optimal myosin—actin overlap will vanish or at least be less prominent by taking sarcomere or half sarcomere non-uniformities into account (Fig. 3B).

**Fig 4:**
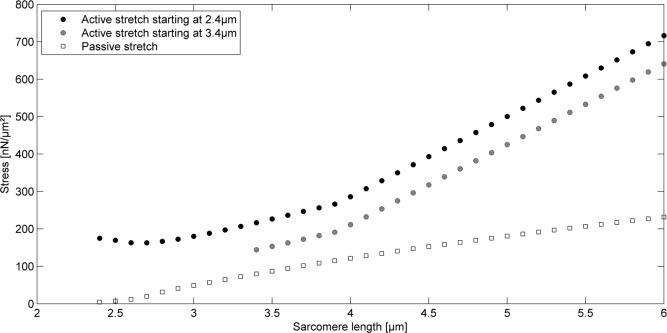
Force-elongation predictions of sarcomeres stretched beyond actin-myosin filament overlap. When a sarcomere is activated at 3.4μm and then stretched actively beyond actin-myosin overlap, its force will exceed the purely passive forces, but will not reach the high forces of sarcomeres stretched actively from optimal length.

Moreover, the model predicts experimentally observed history dependence of force production [2]. Force of a sarcomere that is activated at a length of 3.4μm and then stretched actively until the first protein structures rupture, exceeds the purely passive forces because of the elongation of titin’s free spring length, which is reduced by the length of the proximal IG domains at the time of activation. Due to the passive stretch to 3.4μm before activation, the length of the proximal IG domains is greater than the length of the proximal IG domains at optimal length (2.4μm). Therefore, at a given sarcomere length, the length of the PEVK plus distal IG domains is shorter when the sarcomere is activated at 3.4μm compared to when it is activated at 2.4μm. Hence, the force elongation curve is shifted to higher half sarcomere lengths and half sarcomeres do not reach as high forces as sarcomeres stretched actively from optimal (2.4μm) sarcomere length (Fig. 4).

### Residual force enhancement and force depression

**Fig 5:**
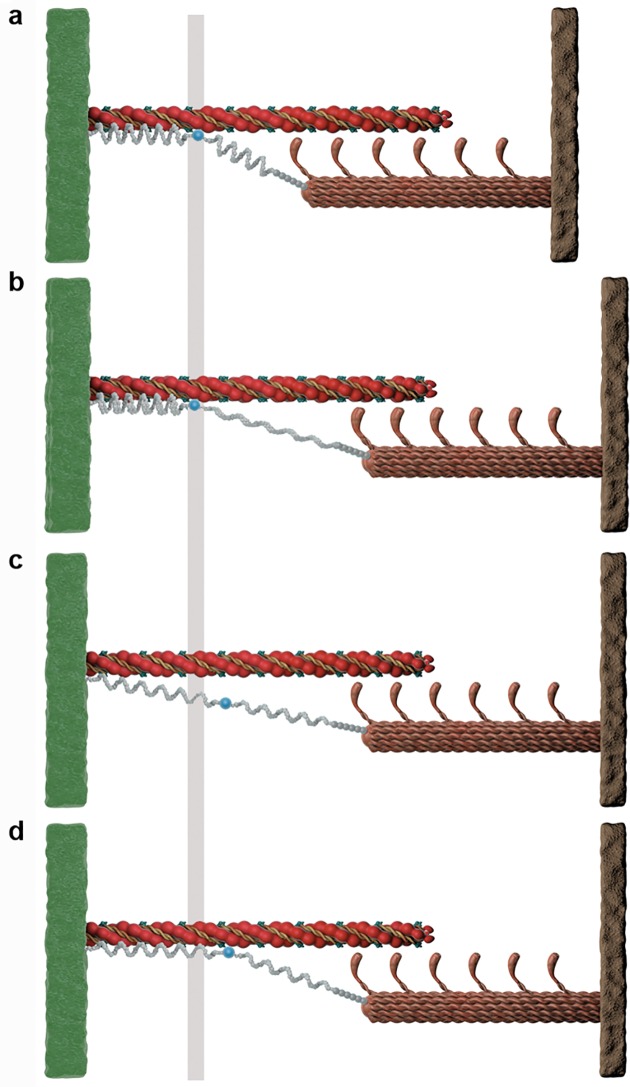
Illustration of an active stretch of a sarcomere based on the three filament model. a, The sarcomere is activated at a length of 2.4μm and b, stretched to 3μm. The resulting force is compared to the corresponding isometric force at 3μm where the sarcomere is activated at a length of 3μm and the elongation of titin’s free spring length is less compared to **b** independent of whether titin is attached to actin **d** or not **c**.

**Fig 6:**
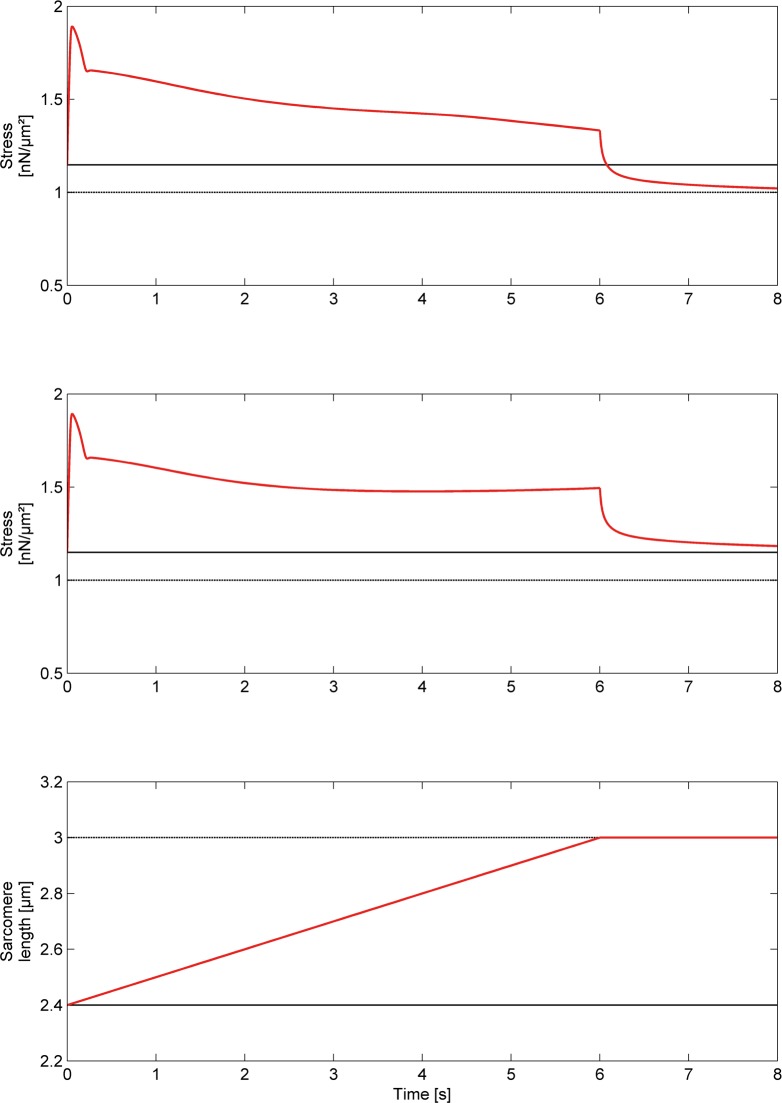
Simulation of an experiment where the sarcomere is activated at a length of 2.4μm and stretched to 3μm (see also Fig. 5). The resulting force is normalized to the corresponding isometric force at 3μm. Forces for the regular cross-bridge model (top panel) and the three filament model (second panel) are calculated for a stretching velocity of 100nm/s/Sarcomere length. The corresponding sarcomere length is shown in the third panel. In contrast to the regular cross-bridge model the three filament model predicts force enhancement in a single (half) sarcomere.

**Fig 7:**
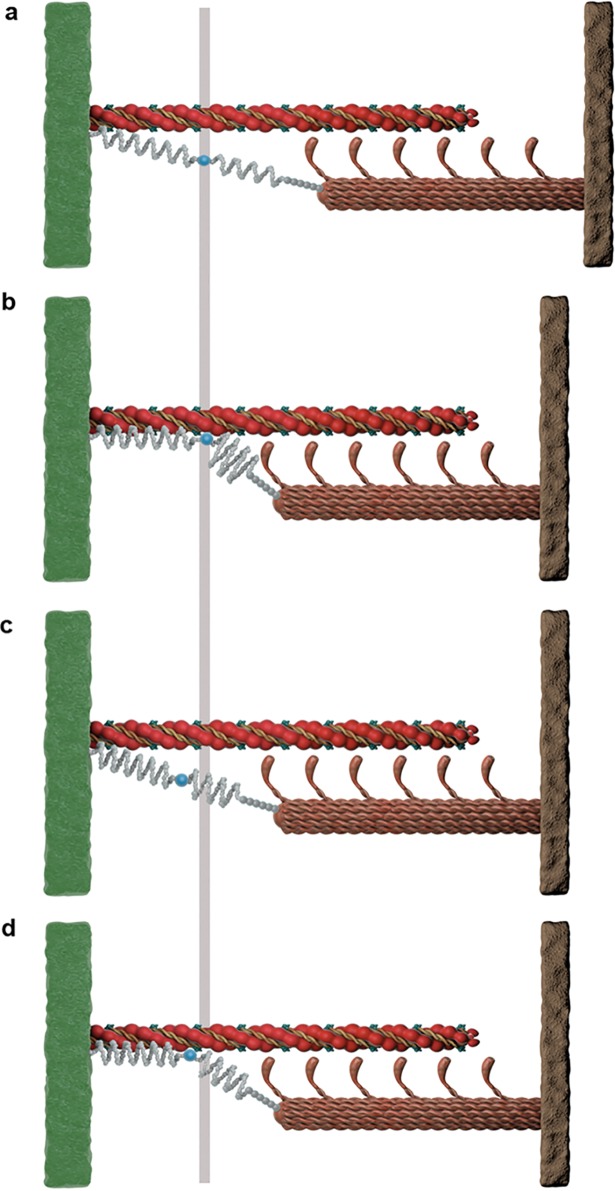
Illustration of active shortening of a sarcomere based on the three filament model. **a**, The sarcomere is passively stretched to a length of 2.6μm, activated and **b**, shortened to 2.4μm. The resulting force is compared to the isometric force at 2.4μm, where the sarcomere is activated at a length of 2.4μm and the elongation of its free spring length is higher, whether titin is attached to actin **d** or not **c**.

**Fig 8:**
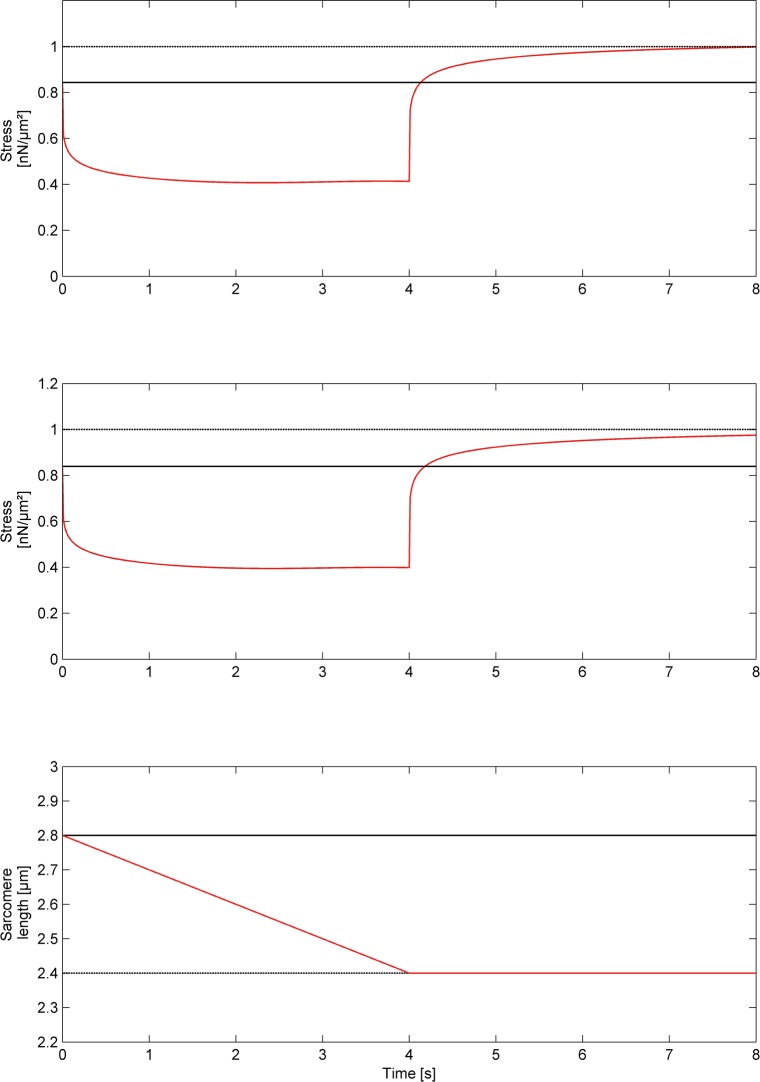
Simulation of an experiment where the sarcomere is passively stretched to a length of 2.6μm, activated and shortened to 2.4μm (see also Fig. 7). The resulting force is normalized to the isometric force at 2.4μm. Forces for the regular cross-bridge model (top panel) and the three filament model (second panel) are calculated for a shortening velocity of 100nm/s/Sarcomere length. The corresponding sarcomere length is shown in the third panel. In contrast to the regular cross-bridge model the three filament model predicts small but significant force depression in a single (half) sarcomere.

We tested the model for a possible contribution to residual force enhancement and force depression in a single half sarcomere [11,46]. In order to elucidate the contribution of actin-titin interactions to enhanced force, we limited our calculation to one (half) sarcomere. Due to the proposed molecular mechanism of force regulation in active stretching and shortening, both residual force enhancement (Fig. 5 and Fig. 6) and force depression (Fig. 7 and Fig. 8) are observed with this model and allow for a simple explanation of these experimentally observed history-dependent effects. Force enhancement starts already during the stretching period. The model predicts higher forces than isometric forces at optimal actin-myosin overlap, as has been observed experimentally for single sarcomere testing [11].

### Passive force enhancement

To test the model’s ability to predict passive force enhancement, we deactivated sarcomeres for the experimental conditions shown in Fig. 6, held the sarcomeres at end length for 5 seconds after which we analysed the forces. As we have no experimental evidence of unbinding rates of titin and actin, or unbinding rates of Calcium from titin, we considered two extremes: first, we assumed that titin does not unbind from the actin filament within the time frame given in experiments and Calcium-induced changes on the titin filament were completely reversed. Then, we assumed that Calcium-induced changes on the titin filament were completely reversed upon deactivation and titin unbinds from the actin filament within the first milliseconds after deactivation. We predict passive force enhancement in both scenarios, i.e. 57% for the first scenario and 37% for the second. Likely, the true behaviour for a sarcomere will lie between the two extreme conditions considered here.

## Discussion

We built a structural three filament model of sarcomere force production to analyse its qualitative behaviour. In order to eliminate any bias toward the three filament model, we refrained from merely fitting existing experimental data, but rather used model parameters from the literature, if they were available. We used parameter estimations for passive force production based on an independent set of data. In order to reproduce experimental data, especially for optimal sarcomere lengths, one might want to fit parameters of the cross-bridge model.

The new model provides an intriguingly simple explanation for force generation in single myofibrils beyond cross-bridge interaction. History dependence of force generation at very long sarcomere length observed in experiments [2] is a straight forward result of the three filament model. Moreover, the three filament model, and specifically the titin behaviour in the model, provides a simple explanation for the force enhancement and force depression properties of skeletal muscles which have been observed for more than half a century without adequate explanation. Various mechanisms have been proposed in the past to explain the enhanced force properties of skeletal muscle. These explanations include the sarcomere length non-uniformity theory [47] or modified cross-bridge kinetic models [48]. However, all previously proposed mechanisms have some serious shortcomings, e.g. [11,21]. The current three filament model overcomes two major shortcomings of these previously proposed theories: first, it predicts residual force enhancement in a single half-sarcomere, thus there is no need for the development of sarcomere or half-sarcomere lengths non-uniformities, and second, it naturally predicts enhanced forces much greater than the isometric reference forces at optimal actin-myosin overlap, a feat not possible with previous models but an experimental observation that has been made on all structural levels of muscle (single sarcomere and myofibril [11], single fibres [49], whole muscle [50,51]). Moreover, the proposed three filament interaction model predicts, in agreement with experimental observations, that force enhancement is greater with higher stretch magnitudes because force generation by titin becomes more prominent for increased stretch magnitudes. Another prediction that agrees well with experimental observations e.g. [11,20,47] is the loss of force enhancement when a sarcomere or muscle is deactivated and then reactivated in the enhanced state. In the three filament model this loss is due to the unbinding of titin from the actin filament during deactivation. Therefore, enhanced forces after reactivation are only those associated with the magnitude of the passive force enhancement.

The difference between force predictions of a classical cross-bridge model and the three filament model is minimal for isometric contractions and concentric contractions. However, metabolic cost for eccentric contractions is small in the three filament model and corresponds to experiments in classical setups.

Force generation is crucially influenced by the choice of the region of titin binding to actin. We assumed that only the proximal IG domains are excluded from titin’s free spring length when titin binds to actin. Since there is a strong body of evidence for the interaction of titin and actin at the PEVK region [15,18,43,52,53], our assumption is rather conservative. If we assumed that some parts of the PEVK region are also bound to actin, we would obtain significantly more prominent model features. For example, forces at long sarcomere lengths would be substantially higher than predicted with the current model, and both, residual force enhancement and force depression would be increased compared to those shown in Fig. 6 and Fig. 8. Therefore, an experimental proof where titin binds to actin, and whether this binding site is variable and depends on the contractile conditions, is essential for accurate force predictions [13].

A challenge of our model is the prediction of titin’s structure at short sarcomere lengths and its spatial distribution relative to actin. As soon as sarcomere lengths become so small that titin is below its slack length, it is mathematically impossible to determine uniquely the length of titin’s individual segments. Therefore, one has to assume titin’s configuration at small sarcomere lengths to predict its free spring length following activation. Experimental results on titin’s segment lengths below slack length are required for accurate predictions of titin’s force contributions when muscles are stretched from relatively short lengths.

Future models should account for attachment and detachment rates of actin-titin interactions and Calcium-induced changes in titin properties. Although these additions to the model will not change the qualitative behaviour demonstrated here, they would be important to quantify the impact of these binding rates on the mechanical properties of muscles and sarcomeres. While the rates of Calcium-induced changes of titin properties might be determined experimentally, it would be difficult to quantify actin-titin interaction rates without a proper model. In addition, as soon as time becomes a major parameter in the model, friction will play a role leading to a stochastic kinetic model that requires thorough analysis.

The three filament model introduced in this paper provides a simple mechanism by which titin contributes to muscle force development, it offers an intuitively appealing explanation of history-dependent phenomena (residual force enhancement and force depression) that have eluded satisfactory explanation based on cross-bridge modelling, and explains mechanistically force generation beyond actin-myosin overlap.
