# Correction: A Novel Three-Filament Model of Force Generation in Eccentric Contraction of Skeletal Muscles

**DOI:** 10.1371/journal.pone.0141188

**Published:** 2015-10-16

**Authors:** The PLOS ONE Staff

In the Funding section, the grant number from the funder Austrian Science Fund (FWF) is missing. The grant number is: T 478-N13. The publisher apologizes for the error.
